# Polyclonal antibodies selectively inhibit tumor growth and invasion and synergize with immune checkpoint inhibitors

**DOI:** 10.1172/jci.insight.166231

**Published:** 2024-02-08

**Authors:** Carine Ciron, Pierre Morice, Juliette Rousse, Patrice Roy, Pierre-Joseph Royer, Olivier Gauthier, Sophie Brouard, Odile Duvaux, Firas Bassissi, Bernard Vanhove

**Affiliations:** 1 Xenothera, Nantes, France.; 2 CRIP, Oniris, INRAE, Nantes, France.; 3 Nantes Université, Inserm, CHU Nantes, Center for Research in Transplantation and Translational Immunology, UMR 1064, ITUN, Nantes, France.

**Keywords:** Oncology, Cancer immunotherapy

## Abstract

Heterologous polyclonal antibodies (pAb) were shown to possess oncolytic properties a century ago with reported clinical responses. More recent preclinical models confirmed pAb efficacy, though their ability to tackle complex target antigens reduces susceptibility to tumor escape. Owing to the recent availability of glyco-humanized pAb (GH-pAb) with acceptable clinical toxicology profile, we revisited use of pAb in oncology and highlighted their therapeutic potential against multiple cancer types. Murine antitumor pAb were generated after repeated immunization of rabbits with murine tumor cell lines from hepatocarcinoma, melanoma, and colorectal cancers. Antitumor pAb recognized and showed cytotoxicity against their targets without cross-reactivity with healthy tissues. In vivo, pAb are effective alone; moreover, these pAb synergize with immune checkpoint inhibitors like anti–PD-L1 in several cancer models. They elicited an antitumor host immune response and prevented metastases. The anticancer activity of pAb was also confirmed in xenografted NMRI nude mice using GH-pAb produced by repeated immunization of pigs with human tumor cell lines. In conclusion, the availability of bioengineered GH-pAb allows for revisiting of passive immunotherapy with oncolytic pAb to fight against solid tumor and cancer metastasis.

## Abstract 2

<p>Tumor cell-targeting glyco-humanized polyclonal&nbsp;antibodies have potential&nbsp;against circulating and&nbsp;solid tumors and synergize&nbsp;with immune checkpoint inhibitors.</p>

## Introduction

For decades, surgery, chemotherapy, and radiotherapy have represented the standard of care for cancer patients. More recently immunotherapy has appeared in the front stage, bringing hope and good results in cancers that are currently without effective care. The mechanism of action of cancer immunotherapy strategies is to activate the immune system against target antigens that are selectively expressed in malignant cells but not in cells of normal tissues. This approach encompasses different kinds of treatments: adoptive cell therapy, cancer vaccines, immunomodulators, oncolytic virus therapy, and targeted antibodies. Although immunotherapy is experiencing a resurgence of interest, the concept is not new and goes back to the late nineteenth century, when infectious agents were used to stimulate immune responses to cancer (1). Immunotherapy reached a turning point in 2011, with clinical approval for antibodies that specifically block CTLA-4 for melanoma and, later on, extended to other immune checkpoints and to many indications. This successful discovery of T cell immune checkpoint was awarded with the 2018 Nobel prize in Physiology or Medecine (2). Two other weapons reinforce the arsenal in immunotherapy and have been approved in clinic: mAbs targeting vascular growth (3, 4) and those directly targeting tumor cells (5). These mAbs are cytotoxic under their native form or are linked to a toxic molecule as antibody-drug-conjugate (ADC) (6). However, by targeting a single specific tumoral antigen, mAbs are susceptible to tumor escape. Indeed, due to their high mutagenic capacity and survival capacities, cancer cells use several mechanisms to evade the host immune response to reestablish their growth and continue to progress (7). Key evasion tactics include upregulation of checkpoint receptor ligands, attraction of immune-suppressing cells, or production of suppressive cytokines. Other specific mechanisms include downregulating the facets of the antigen presentation system or the antigen itself. In lung cancer, patients relapse under anti-EFGR therapy due to the appearance of a mutation in EGFR (T790M) (8). In colorectal cancer, genetic alterations confer a selective advantage to tumor cells when under the pressure of anti-EGFR therapy (9). Another example applies when patients with NY-ESO-1^+^ myeloma targeted with a CAR-T have developed recurrences caused by tumor escape (10). There is, therefore, a need to target multiple antigens to improve tumor escape. Building upon the recent therapeutic success of bispecific antibodies, and supported by accelerating progress in genetic engineering methods, the field of multispecific therapeutic antibodies is growing rapidly and can dramatically change cancer treatment landscape (11).

A broad variety of multitarget antibody formats has been developed to function through different mechanisms in cancer immunotherapy. One of these different approaches includes targeting multiple tumor antigens or different antigen epitopes on tumor cells to increase tumor selectivity (12, 13).

Targeting multiple tumor antigens with oncolytic pAb has demonstrated their efficacy initially in humans, more than a century ago, when Héricourt and Richet used sera from immunized animals to treat children with sarcomas (14). Then, multiple preclinical cancer models confirmed the oncolytic activity of pAb (15–20). Their mechanism of action combines complement activation (complement-dependent cytotoxicity [CDC]) and cascade activation of effector T lymphocytes (21), recruitment of effector cells (antibody-dependent cellular cytotoxic [ADCC], antibody-dependent cellular phagocytosis [ADCP]), direct induction of apoptosis, and epitope masking, resulting in inhibition of molecular interactions. The mechanism of action of pAb has been best demonstrated for thymoglobulin, a polyclonal antilymphocyte serum (22). By targeting multiple epitopes, pAb minimize the emergence of variants that can escape treatment. These preclinical data have motivated several international teams to consider pAb as a potentially new therapeutic tool in oncology (23), especially since the development of bioengineered pAb limited adverse effects due to administration of heterologous animal-derived immunoglobulins such as serum sickness disease (SSD) in humans (24). For instance, pigs have been genetically engineered to knock out the cytidine monophosphate-N-acetylneuraminic acid hydrolase (CMAH) and α1,3-galactosyl-transferase (GGTA1) (25), the 2 main xenogenic glycoantigens responsible for animal anti-IgG responses in humans. Immunoglobulins form these animals are devoid of Neu5Gc and αGal carbohydrate epitopes and therefore exhibit low immunogenicity in human (26, 27).

In this study, we first produced 3 rabbit polyclonal antibodies (pAb) against murine tumors: antimelanoma, antihepatocellular carcinoma, and anticolorectal cancer. Their reactivity against tumor and parental tissues was evaluated, and they were used alone or in combination with immune checkpoint inhibitors in an orthotopic syngeneic hepatocellular carcinoma (HCC) model, a heterotopic syngeneic colorectal carcinoma (CRC) model, and a heterotopic syngeneic melanoma model (SKCM). The concept was then applied to human tumors using melanoma, colon adenocarcinoma, and hepatocarcinoma cancer cell lines. Antitumor Glyco-humanized pAb (GH-pAb) were generated using double KO pigs knocked out for CMAH and GGTA1 and selected for the absence of tissue cross-reactivity against healthy tissues.

## Results

### pAb promote target cancer cells lysis, without off-target toxicity on healthy cells in vitro.

**Figure 1:**
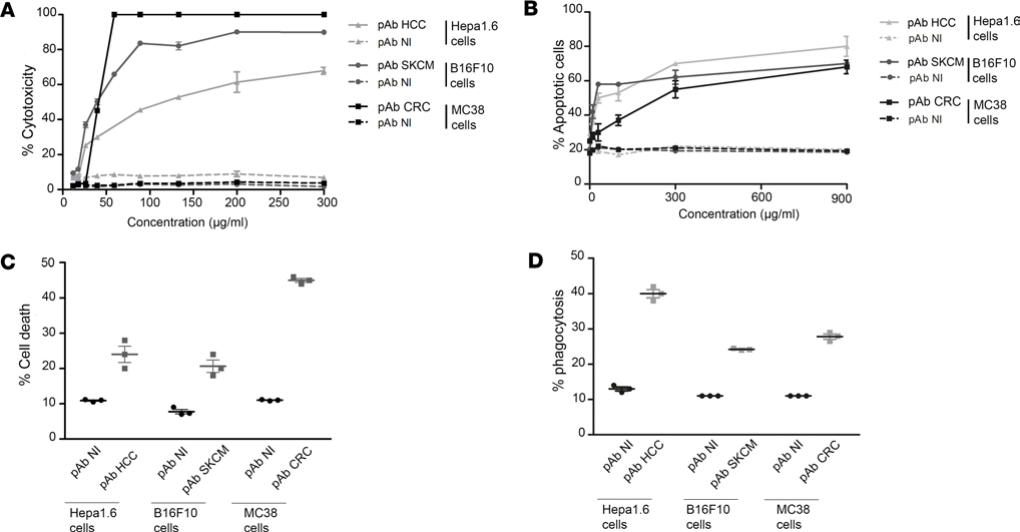
Oncolytic polyclonal antibodies promote target cancer cells lysis by CDC, apoptosis, ADCC, and ADCP. (**A**) CDC assay by incubating various concentrations of antitumor pAb, nonimmune pAb, and rabbit complement with target cancer cells. Cell lysis was then measured by incorporation of propidium iodide by flow cytometry (*n* = 3). (**B**) Apoptosis on target cancer cells after incubation with antitumor pAb and nonimmune pAb during 3 hours at 37°C (*n* = 3). (**C**) Murine cancer cell lines were cocultured with murine NK cells and 5 μg/mL of antitumor and nonimmune pAb for 16–24 hour and then analyzed for cell deaths (*n* = 3). (**D**) Murine cancer cell lines were cocultured with murine macrophages and 5 μg/mL of antitumor and nonimmune pAb for 3 hours; then, phagocytosis was assessed as the percentage of double-positive cells (CFSE^+^/F4/80^+^) (*n* = 3). All data are expressed as mean ± SEM.

Three batches of pAb were developed by hyperimmunization of rabbits with murine cancer cells derived from murine HCC, murine melanoma (SKCM) and murine colon adenocarcinoma (CRC). Their ability to promote target cancer cell lysis by CDC, apoptosis, ADCC, and ADCP was evaluated in cancer cell lines representative of the targeted cancer (Hepa1.6 for HCC, B16F10 for and MC38 for CRC). IgG anti-HCC (pAb HCC), IgG anti-B16F10 (pAb SKCM), and IgG anti-CRC (pAb CRC) displayed dose-dependent CDC activity against their targets (up to 100% of cytotoxicity), while no CDC was observed with nonimmune rabbit IgGs (Figure 1A). The maximal CDC activity was obtained at 200 μg/mL with pAb HCC, from 90 μg/mL with pAb SKCM and from 60 μg/mL with pAb CRC. The 3 oncolytic pAb also displayed dose-dependent apoptotic activity against their targets (Figure 1B), while no apoptosis was observed with nonimmune rabbit IgGs. They also demonstrated a strong ADCC ranging from 20% to 45% of cell death depending on the pAb, at a concentration of 5 μg/mL (Figure 1C), and a strong ADCP ranging from 24% to 40% depending on the antitumor pAb, at a concentration of 5 μg/mL (Figure 1D).

**Figure 2:**
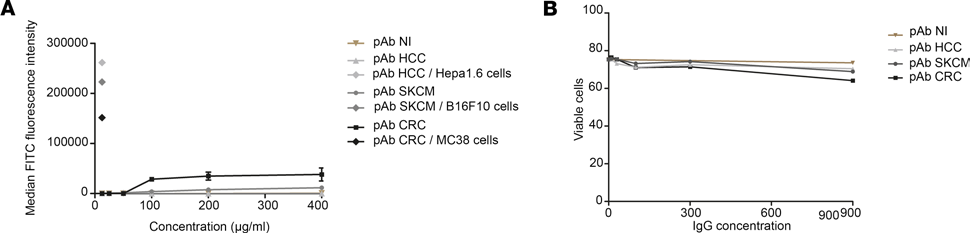
Oncolytic polyclonal antibodies do not crossreact with normal murine splenocytes. (**A**) Murine normal cells (splenocytes) were incubated at 4°C with various concentrations of anticancer pAb and nonimmune pAb. Detection of bind pAb was performed with FITC-conjugated secondary antibodies (anti-rabbit). Binding was then measured by flow cytometry. The same experiment is performed with cancer cells and anticancer pAb as a positive control (*n* = 3). (**B**) Apoptotic activity of anticancer pAb on murine normal cells is evaluated by using murine splenocytes (*n* = 3). All data are expressed as mean ± SEM.

We then analyzed their capacity for recognition, binding, and possible apoptotic activity on normal cells (splenocytes of mice). pAb HCC, pAb CRC, pAb SKCM, and nonimmune rabbit pAb did not show binding to murine normal splenocytes (Figure 2A) and did not induce apoptotic activity on these cells (Figure 2B). pAb CRC displayed a slight binding to murine splenocytes from 100 μg/mL, with no increase at higher concentration (Figure 2A). This slight binding was not translated into apoptotic activity on healthy murine cells (Figure 2B). To confirm the absence of reactivity on healthy tissue, we performed a tissue cross-reactivity study in several murine tissues by IHC in heart, lung, CNS, spinal cord, liver, and striated muscle by incubating IgG at the optimum staining concentration, as determined during method development. All tissue sections were evaluated for the distribution and intensity of staining. This analysis revealed no staining of the different oncolytic pAb on analyzed healthy murine tissues, suggesting the absence of significant cross-reactivity (Supplemental Figure 1; supplemental material available online with this article; https://doi.org/10.1172/jci.insight.166231DS1).

**Figure 3:**
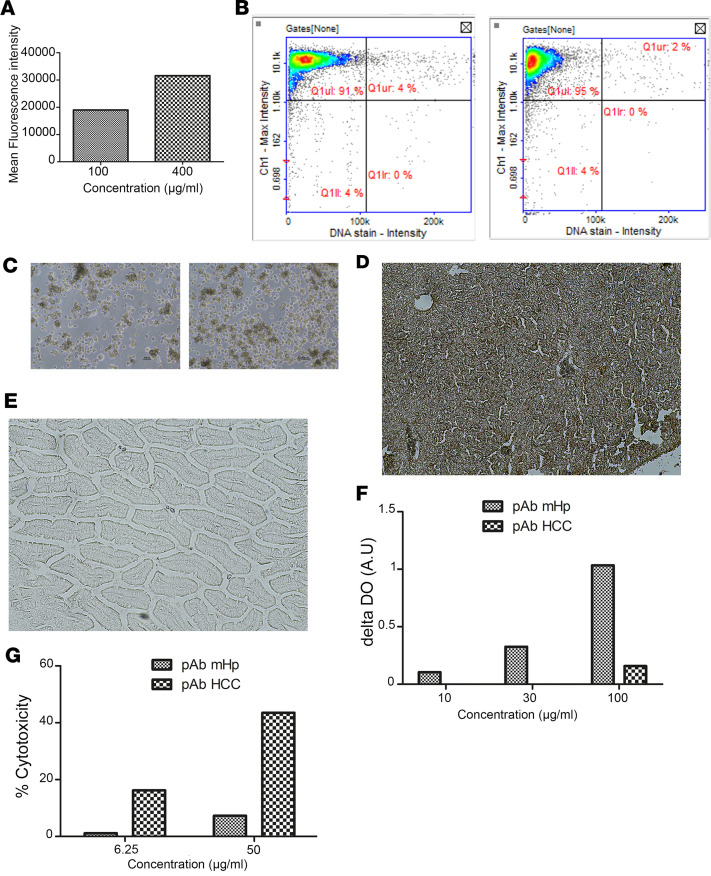
Polyclonal antibodies raised against healthy cells show high toxicity against normal tissues with low antitumor activity. (**A**) Binding of pAb mHp at 2 concentrations (100 μg/mL and 400 μg/mL) on healthy murine primary hepatocytes. (**B**) Plots demonstrating the labeling of all hepatocytes at 100 μg/mL (left plot) and at 400 μg/mL (right plot). (**C**) Photomicrographs of hepatocytes culture. (**D**) Photomicrograph showing immunostaining with pAb mHp at a concentration of 5 μg/mL on a section of liver from a healthy mouse (brown). (**E**) Photomicrograph showing the absence of immunostaining with pAb mHp at a concentration of 5 μg/mL on a section of intestine from a healthy mouse. (**F**) LDH assay showing an increase in LDH associated with an increase in pAb mHp concentration, indicating cytotoxicity in healthy hepatocytes. In contrast, no increase in LDH was observed after incubation with pAb HCC (*n* = 2). (**G**) Complement-dependent cytotoxicity showing antitumor toxicity in Hepa 1.6 cells with pAb HCC and not with pAb mHp (*n* = 2). All data are expressed as mean ± SEM. Scale bars: 50 µm.

In order to determine whether the antitumor activity of our pAb is indeed characteristic of polyclonal antitumor antibodies, we generated pAb directed against healthy mouse primary hepatocytes (pAb mHp). As expected, pAb mHp exhibited strong binding to murine hepatocytes (Figure 3, A–C). In addition, IHC also showed strong labeling of a section of liver from healthy mice (Figure 3 D), and no tissue cross-reactivity on a section of intestine (Figure 3E). We then assessed the complement-dependent cytotoxic activity of pAb mHp in comparison with pAb HCC on healthy murine hepatocytes or Hepa1.6 cells. Only pAb mHp showed cytotoxic activity against healthy hepatocytes, as demonstrated by the increase in LDH release from these cells when subjected to increasing concentrations of the polyclonal antibody (10, 30, and 100 µg/mL) (Figure 3F). In contrast, Hepa1.6 tumor cells were weakly targeted and killed by pAb mHp. Concentrations of polyclonal antibodies ranging from 6.25 to 50 μg/mL were evaluated, and no cytotoxicity was observed on Hepa1.6 cells until a concentration of 50 µg/mL of pAb mHp was reached, whereas cytotoxicity was already observed with the pAb HCC antibody from 6.25 μg/mL (20% cell death) (Figure 3G).

### pAb can promote destruction of the tumor, delay its growth, or synergize with anti–PD-L1 antibodies.

We evaluated the therapeutic efficacy of these 3 pAb in syngeneic murine models representative of the following cancers: a HCC with Hepa1.6 cells, a colon adenocarcinoma with MC38 cells, and a melanoma with B16F10 cells. pAb were evaluated in monotherapy and in association with anti–PD-L1 antibodies.

**Figure 4:**
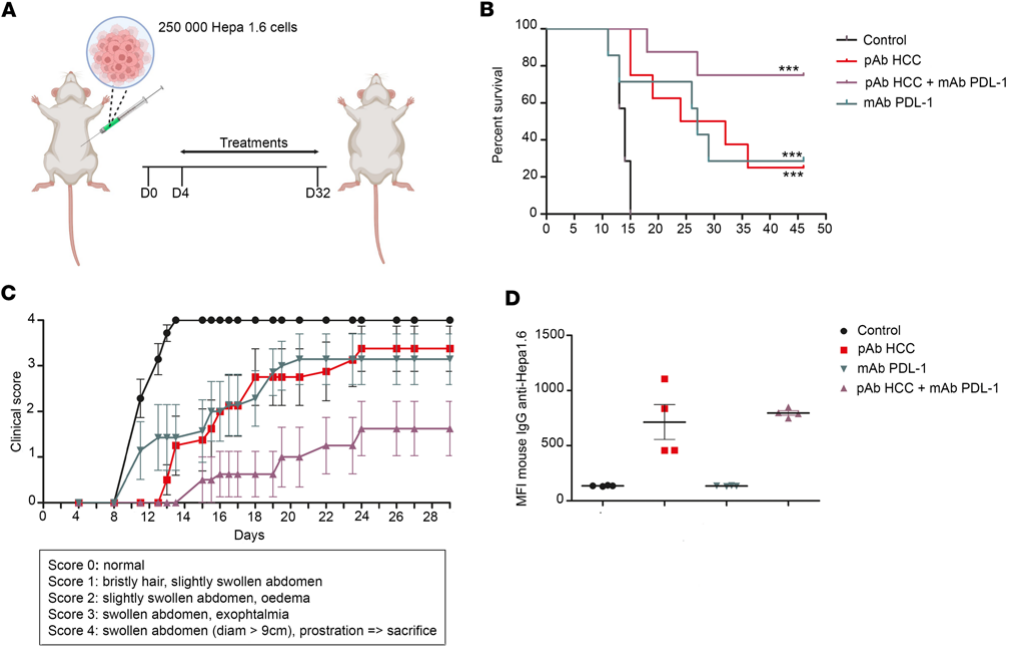
Efficacy of anti–hepatocellular carcinoma pAb, alone or in association with anti–PD-L1, in a syngeneic orthotopic in vivo mouse model of hepatocellular carcinoma. (**A**) Schematic of treatment strategy for syngeneic mouse model. (**B**) Kaplan-Meier curve; mice were treated twice a week from day 4 after tumor cell injection and during 4 weeks with: control isotype 3G8 at 5 mg/kg and nonimmune rabbit pAb at 12.5 mg/kg (group “control”, *n* = 7); control isotype 3G8 at 5 mg/kg and pAb HCC at 12.5mg/kg (group “pAb HCC”, *n* = 8); mAb PD-L1 at 5 mg/kg and pAb HCC at 12.5mg/kg (group “pAb HCC + mAb PD-L1, *n* = 8); mAb PD-L1 at 5 mg/kg and nonimmune rabbit pAb at 12.5 mg/kg (group “mAb PD-L1”, *n* = 7). (**C**) Evolution of the cancer clinical score in mice. (**D**) Sera collected from live mice at the end of the experiment (day 46) are evaluated for the presence of natural murine IgG anti-HCC. ***P < 0.001; log-rank test, χ^2^ test, and 1-way ANOVA post hoc test Newman-Keuls*.* All data are expressed as mean ± SEM.

In the HCC, murine tumors were induced in liver by intraportal injection of tumor cells. Treatment was initiated from day 4 after surgery. Animals were dosed i.p. twice a week for a total duration of 28 days (Figure 4A) and followed up until day 48. A clinical score from 1 to 4 was assigned, with the higher score of 4 triggering euthanasia, for ethical reasons. Resulting scores and mortality rates are shown in Figure 4, B and C. Mice from the control treatment group displayed a maximal clinical score as early as day 15 with no survival. Mice dosed with either pAb HCC or anti–PD-L1 monotherapies showed an intermediate profile with a progressive increase of the median clinical score from day 8 to day 35, stabilizing at the end of the protocol with 30% survival. Mice receiving the anti–PD-L1 antibody in combination with pAb HCC had a survival rate of 75% at day 48 and a median clinical score of 1.5, indicating a synergy of action. Interestingly, surviving mice (at day 46) receiving pAb HCC (alone or with anti–PD-L1), but not mice treated with anti–PD-L1 alone, mounted a murine anti-HCC IgG antibody response in their serum, indicating that the tumor had been made visible for the adaptative mouse immune system (Figure 4D). Control mice at euthanasia (<day 15) did not develop antitumor antibodies.

**Figure 5:**
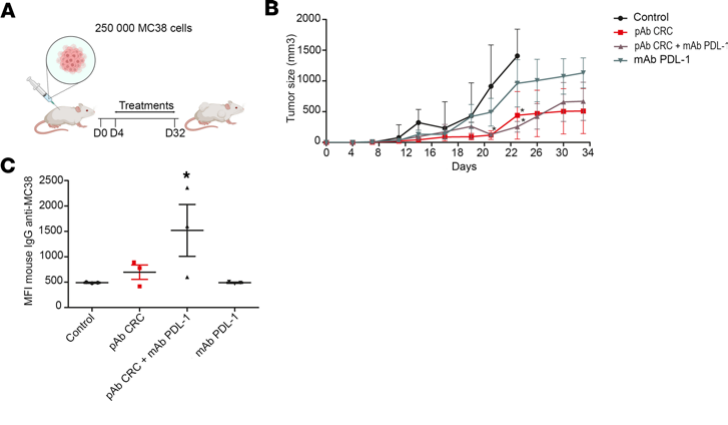
Anticolon adenocarcinoma pAb, alone or in association with anti–PD-L1, slow down tumor growth. (**A**) Schematic of treatment strategy for syngeneic mouse model. (**B**) Tumor growth evolution; mice were treated twice a week from day 4 after tumor cells injection and during 4 weeks with: control isotype 3G8 at 5 mg/kg and nonimmune rabbit pAb at 12.5mg/kg (group “control”, *n* = 7); control isotype 3G8 at 5 mg/kg and pAb CRC at 12.5mg/kg (group “pAb CRC), *n* = 8); mAb PD-L1 at 5 mg/kg and pAb CRC at 12.5mg/kg (group “pAb CRC + mAb PD-L1”, *n* = 8); and mAb PD-L1 at 5 mg/kg and nonimmune rabbit pAb at 12.5 mg/kg (group “mAb PD-L1”, *n* = 7). (**C**) Sera collected from live mice at the end of the experiment (day 38) are evaluated for the presence of natural murine IgG anti-MC38. *P < 0.05; 1 way ANOVA post hoc test Newman-Keuls and Fisher’s exact test. All data are expressed as mean ± SEM.

In the s.c. colon adenocarcinoma model, animals were treated twice a week for 32 days and followed up until day 40 (Figure 5A). Tumor growth was evaluated by tumor volume measurement (Figure 5B). When s.c. tumor reached 1,500 mm^3^, euthanasia was triggered for ethical purpose. Mice from the control group displayed a rapid increase of the tumor size from day 7, with all mice reaching the end point (tumor volume of 1,500 mm^3^) between day 25 and day 29. Mice treated with anti–PD-L1 antibodies alone showed a relatively slower progressive increase of the median tumor size from day 7 to day 29; this increase was lower than in the control group but without a dramatic change in the end point, as all mice reached the end point at day 29. Mice treated with pAb CRC, either alone or in association with anti–PD-L1, had drastically slower evolution of the tumor size with a median below 400 mm^3^ at day 40 for both groups. The administration of pAb CRC alone was sufficient to slow down tumor growth without additional contribution of mAb PD-L1 on the tumor size. Interestingly, similar to the HCC model, we also recorded an adaptive murine IgG response in mice treated with pAb CRC (Figure 5C), with higher levels of anti-CRC mouse IgG elicited in animals who received the bitherapy.

**Figure 6:**
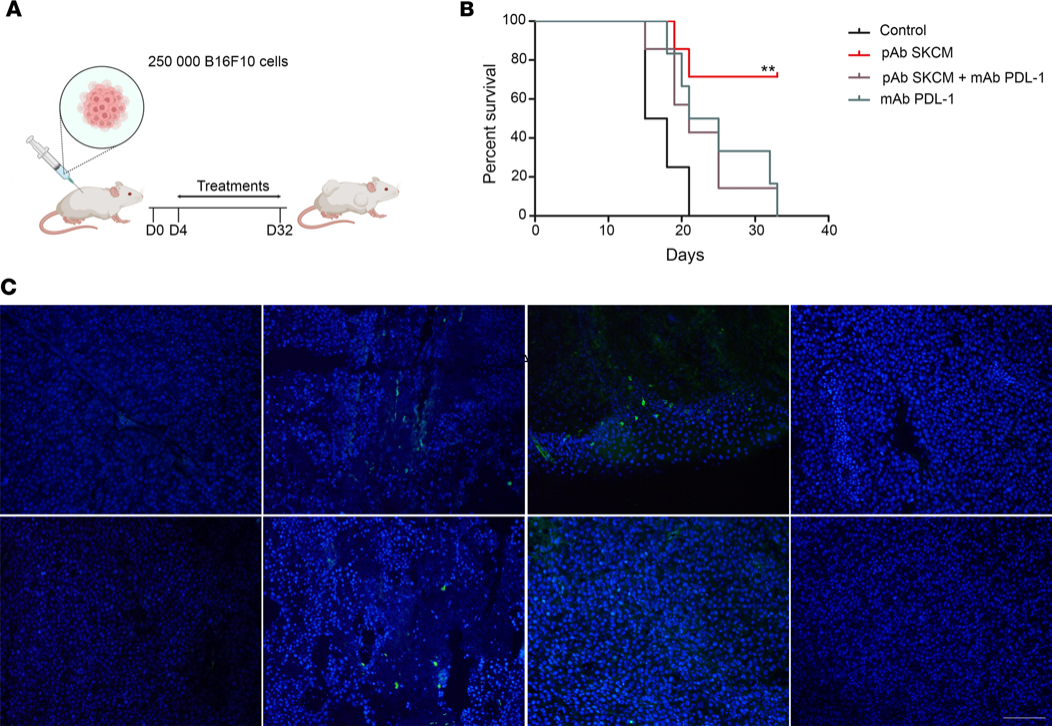
Antimelanoma pAb increase survival rate in a melanoma mouse model and elicit T cells host immune response. (**A**) Schematic of treatment strategy for syngeneic mouse model. (**B**) Kaplan-Meier curve; mice were treated twice a week from day 4 after tumor cell injection and during 4 weeks with: control isotype 3G8 at 5 mg/kg and nonimmune rabbit pAb at 12.5mg/kg (group “control”, *n* = 7); mAb PD-L1 at 5 mg/kg and pAb SKCM at 12.5 mg/kg (group “pAb SKCM”, *n* = 8); mAb PD-L1 at 5 mg/kg and pAb SKCM at 12.5 mg/kg (group “pAb SKCM + mAb PD-L1), *n* = 8); mAb PD-L1 at 5 mg/kg and nonimmune rabbit pAb at 12.5 mg/kg (group “mAb PD-L1”, *n* = 7). **P < 0.01; log-rank test – χ^2^ test. (**C**) Fluorescence IHC showing T cells infiltrates. CD3^+^ and CD8^+^ cells are labeled with a secondary antibody Alexa Fluor 488 (green) and counterstained with DAPI (blue). Scale bar: 50 μm.

In the B16F10 s.c. melanoma model, treatment was initiated from day 4 after surgery and maintained twice a week for 28 days (Figure 6A). Survival was recorded over 32 days (Figure 6B). The median survival was 16.5 days in the control group versus 21 and 23 days in groups receiving mAb PD-L1 in mono- and bitherapy, respectively. Mice treated with pAb SKCM alone did not reach the median survival at the end of experiments, with 70% of animals still alive. In this group, IHC of tumor biopsies was performed on day 45 and showed CD3^+^ T cells and CD8^+^ T cells inflitrates only in groups treated with pAb SKCM in mono- or bitherapy (Figure 6C). It should be noted that tumors from treated groups with mAb PD-L1 were necrotic and ulcerated (data not shown), leading to anticipated sacrifice for ethical purpose.

### Cross-antitumor activity of the anti-CRC colon adenocarcinoma pAb against 4T1 breast cancer cell.

**Figure 7:**
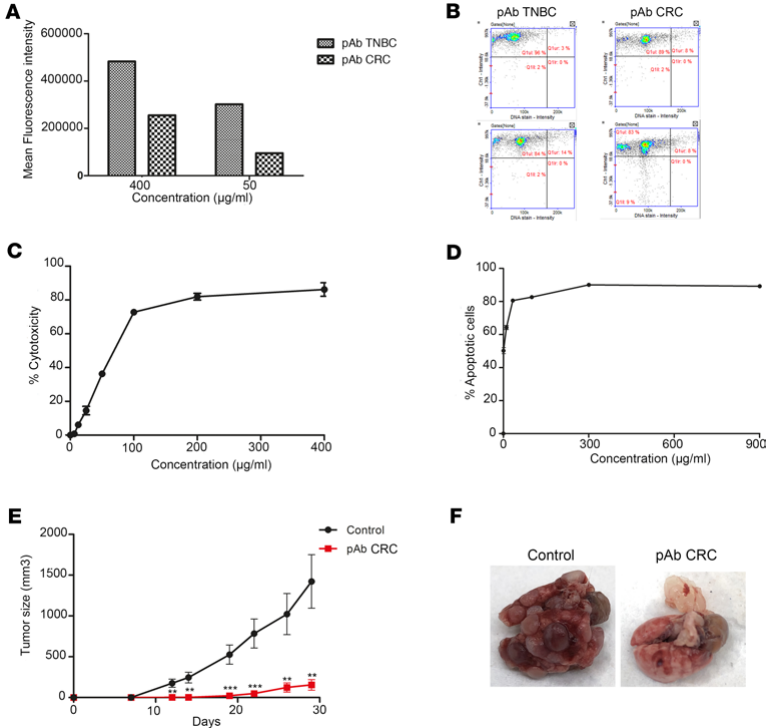
Efficacy of anticolon adenocarcinoma pAb CRC in a syngeneic orthotopic in vivo mouse model of triple-negative breast cancer. (**A**) Binding of various concentrations of pAb TNBC and pAb CRC on 4T1 cells. (**B**) Plots demonstrating the labeling of all 4T1 cells at 400 μg/mL (top plots) and at 50 μg/mL (bottom plots) with pAb. (**C**) Complement dependent cytotoxicity obtained after incubation with serial concentrations of pAb CRC on 4T1 cells compared with pAb (*n* = 3). (**D**) Apoptosis on target cancer cells after incubation with pAb CRC during 3 hours at 37°C compared with pAb NI. (**E**) Tumor size curves; mice were treated twice a week from day 4 after tumor cells injection and during 4 weeks with: no treatment (control, *n* = 10) and pAb CRC at 12.5 mg/kg (*n* = 10). **P < 0.01, ***P < 0.001; Student’s *t* test. (**F**) Lung aspects at the end of the protocol (day 29) showing metastatic nodules for untreated mice. All data are expressed as mean ± SEM.

We observed that the pAb CRC against colon adenocarcinoma cells had the capacity to bind to the breast cancer cell line 4T1 (Figure 7, A and B) and to induce CDC (Figure 7C) and apoptosis (Figure 7D). In a syngeneic orthotopic in vivo mouse model of 4T1 breast cancer, pAb CRC fully controlled 4T1 breast cancer tumor growth until day 19 and then repressed tumor growth, maintaining a size below 160 mm^3^ until the end of the protocol (day 29). In contrast, mice from the control group displayed a rapid increase of the tumor size from day 7, with median tumor size reaching 1,000 mm^3^ from day 26 (Figure 7E). At the end of the protocol (day 29), lungs of control untreated mice visually contained many metastases, whereas mice from the treated group receiving pAb CRC did not display any metastasis (Figure 7F).

**Figure 8:**
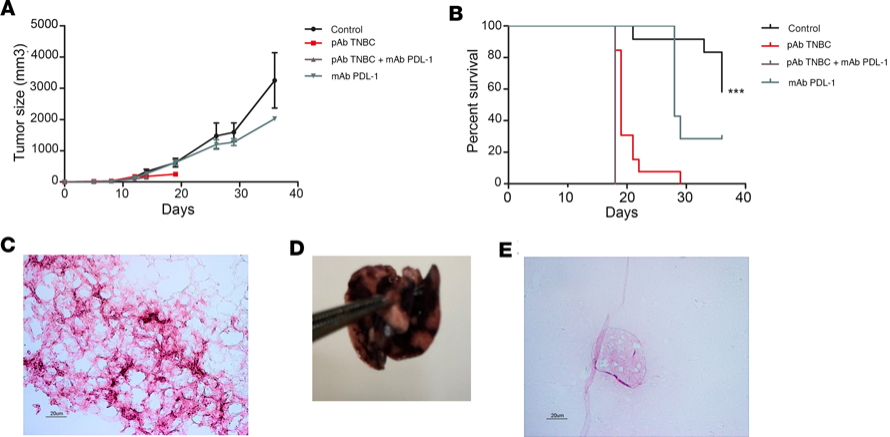
Immunogenicity of antibreast pAb (pAb TNBC) in an orthotopic in vivo mouse model of breast cancer. (**A**) Tumor growth evolution; mice were treated twice a week from day 4 after tumor cells injection and during 4 weeks with: control isotype 3G8 at 5 mg/kg and nonimmune rabbit pAb at 12.5 mg/kg (group “control”, *n* = 7); control isotype 3G8 at 5 mg/kg and TNBC pAb at 12.5 mg/kg (group “TNBC pAb”, *n* = 8); mAb PD-L1 at 5 mg/kg and TNBC pAb at 12.5 mg/kg (group “TNBC pAb + mAb PD-L1”, *n* = 8); and mAb PD-L1 at 5 mg/kg and nonimmune rabbit pAb at 12.5 mg/kg (group “mAb PD-L1”, *n* = 7). Mice from the “TNBC pAb” group and the “TNBC pAb + mAb PD-L1” group all died prematurely, between days 12 and 18. (**B**) Kaplan-Meier curve. ***P < 0.001 log-rank test *–* χ^2^ test. (**C**) Photomicrograph showing immunostaining pAb 4T1 on healthy mouse lung section (pink/purple). (**D**) Lung of treated mice showing pulmonary hemorrhages at autopsy. (**E**) Photomicrograph showing immunostaining TNBC pAb on healthy striatum section (pink/purple). All data are expressed as mean ± SEM.

In order to generate a comparator pAb, we immunized rabbits with a murine breast cancer tumor line (pAb triple negative cancer [TNBC]). When administered to mice in a 4T1 xenograft model, the mice showed significant respiratory distress and neurological impairment within the second week of treatment. This toxicity was only observed in mice that received pAb TNBC (Figure 8, A and B). Neurological damage could be demonstrated by the clasping reflex test. Indeed, mice treated with pAb TNBC exhibited paw-clasping and a bat-like posture. These phenotypes are observed in mice with lesions in cerebellum, basal ganglia, and neocortex (28). After autopsy, we observed that the treated mice presented important pulmonary hemorrhages (Figure 8D). To decipher this toxic effect, we performed IHC of pAb TNBC on healthy mouse tissues. We observed, in vitro, a strong recognition and staining of healthy mouse lung sections by pAb TNBC by IHC (Figure 8C). We also demonstrated recognition and staining in the striatum on normal mouse brain tissue sections (Figure 8E). Lung and basal ganglia in the brain were the only targeted tissues (data not shown), confirming macroscopic observations at autopsy. One of the targets recognized by pAb TNBC is the metabotropic Glutamate receptor 1 (Supplemental Figure 2). This target, which is highly overexpressed in triple-negative breast cancer (29–32), is also highly expressed in striatal neurons (33, 34) and lungs, corresponding to the labeling of these tissues in IHC.

### pAb can abolish metastasis in a pulmonary metastasis model.

**Figure 9:**
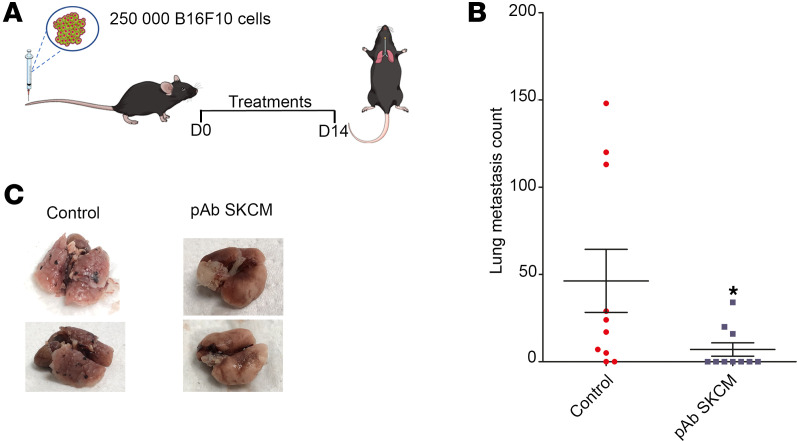
Efficacy of antimelanoma pAb (pAb SKCM) in a syngeneic in vivo mouse model of pulmonary metastasis. (**A**) Schematic of treatment strategy for metastatic pulmonary mouse model. (**B**) Lung metastasis count at day 14. *P < 0.05; Student’s *t* test. (**C**) Lung aspects at the end of the protocol (day 14).

To further investigate the potency of oncolytic pAb to prevent metastasis, we developed a pulmonary metastasis mouse model elicited by i.v. injection B16F10 murine melanoma cells. Treatment was initiated at day 1 after surgery and maintained twice a week for 14 days (Figure 9A). At day 14, mice were sacrificed, and lungs were observed to detect the presence of nodules, easily identifiable by the presence of melanin. Mice from the control group displayed an average of 46 black nodules/metastases on day 14 (Figure 9, B and C), while mice receiving the pAb anti-SKCM presented only 7 nodules, on average (*P* < 0.05), with 7 of 10 mice displaying no nodule at all, indicating efficacy of the treatment with pAb against metastasis and tumor invasion.

### GH-pAb can efficiently decrease tumoral growth in a xenograft mice model.

In order to investigate the efficacy of antitumor pAb developed from our platform of GH-pAb (26), 3 batches of GH-pAb were developed by hyperimmunization of double-KO pigs with human cancer cell lines: a colon adenocarcinoma, an HCC, and a melanoma cell lines. We evaluated their antitumor activity in representative mouse xenograft models.

**Figure 10:**
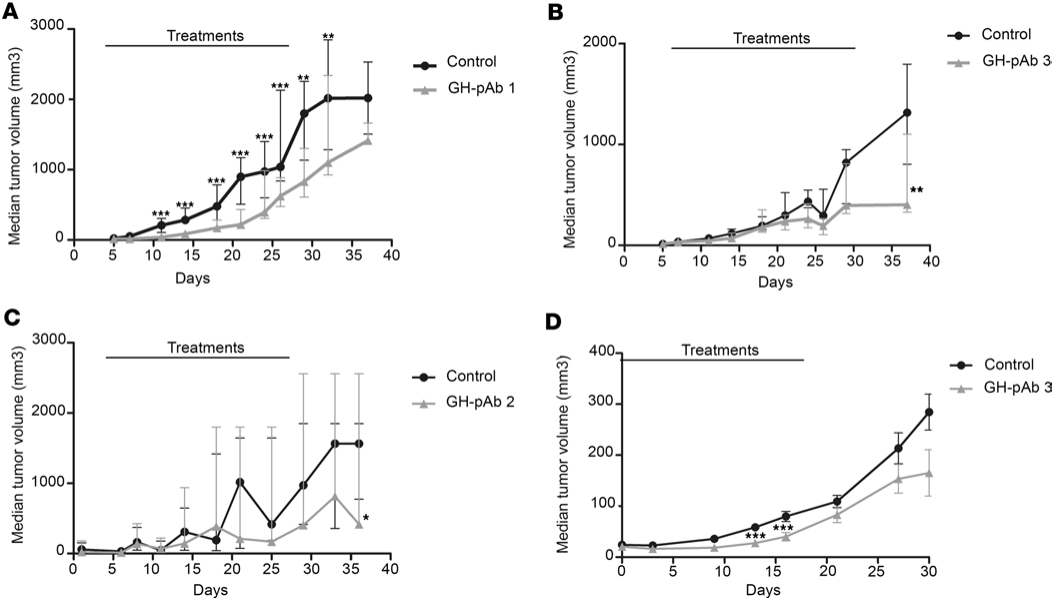
Efficacy of oncolytic GH-pAb in human xenograft tumors model. (**A**) Tumor growth evolution in a mouse model of human colon adenocarcinoma HCT116. Treatment began when tumor size reached 50 mm^3^ and occurred twice a week for 4 weeks. Two groups were included in this study: no treatment (control, *n* = 10); GH-pAb1 at 35 mg/kg (*n* = 10). **P < 0.01, ***P < 0.001; Student’s *t* test. (**B**) Tumor growth evolution in a mouse model of human melanoma SK-Mel-30. Treatment began when tumor size reached 50 mm^3^ and occurred twice a week for 4 weeks. Two groups were included in this study: no treatment (control, *n* = 10); GH-pAb3 at 35 mg/kg (*n* = 10). **P < 0.01; Student’s *t* test. (**C**) Tumor growth evolution in a mouse model of human hepatocellular carcinoma HEPG2. Treatment began when tumor size reached 50 mm^3^ and occurred twice a week for 4 weeks. Two groups were included in this study: no treatment (control, *n* = 10); GH-pAb2 at 35 mg/kg (*n* = 10). *P < 0.05; Student’s *t* test. (**D**) Tumor growth evolution in a mouse model of human NSCLC A549. Treatment with GH-pAb3 began when tumor size reached 50 mm^3^ and occurred twice a week for 4 weeks. Two groups were included in this study: no treatment (control, *n* = 10); GH-pAb3 at 35 mg/kg (*n* = 10). *P < 0.05, ***P < 0.001; Student’s *t* test.

In the HCT116 (colon adenocarcinoma) s.c. xenograft model, treatment with GH-pAb1 was initiated when tumor size reached 50 mm^3^, and it was maintained twice a week for 28 days (Figure 10A). Mice from the control group displayed a rapid growth of the tumor size from day 7, with a median tumor size reaching 1,000 mm^3^ from day 20 and 2,000 mm^3^ at the end of the protocol (day 38). GH-pAb1 efficiently decreased tumor growth from the second injection onward (*P* < 0.001). Treated mice displayed a significant lower tumor growth until day 32 compared with the control group (Figure 10A).

GH-pAb2 was evaluated in the corresponding HCC xenograft model. Treatment was initiated when tumor size reached 50 mm^3^, and it was maintained twice a week for 28 days. Mice from the control group displayed a rapid increase of the tumor size from day 18, with median tumor size reaching 1,700 mm^3^ at the end of the protocol (day 40). Treated mice displayed significantly slower tumor growth, staying below 1,000 mm^3^ at the end of the protocol (day 40) (Figure 10C).

GH-pAb3 was evaluated in a melanoma s.c. xenograft model. Treatment was initiated when tumor size reached 50 mm^3^, and it was maintained twice a week for 28 days. Mice from the control group displayed a rapid increase of the tumor size from day 7, with tumor size reaching 1,300 mm^3^ at the end of the protocol (day 38). Treated mice displayed slow tumor growth, staying below 500 mm^3^ at the end of the protocol (day 38) (Figure 10B).

### GH-pAb exhibit antitumoral activity against multiple cancers without toxicity on healthy tissues.

In order to investigate whether the cross-cancer activity observed in mouse syngeneic models could be confirmed with GH-pAb, we analyzed them by IHC on a tissue microarray including 13 sections of tumor tissues and 13 corresponding sections of healthy tissues. Results are summarized in Table 1. GH-pAb1 was able to bind several tumoral tissues (breast, colon, muscle, liver, lung, pancreas, prostate, skin, intestine, stomach, ovary) and showed cross-reactivity for some healthy tissues (breast, colon, liver, stomach). GH-pAb3 was also able to bind several tumoral tissues (muscle, lung, pancreas, prostate, skin, intestine, stomach), with no cross-reactivity for their corresponding healthy tissues. GH-pAb2 was also able to bind tumoral tissues (liver, lung, ovary), with no cross-reactivity for their corresponding healthy tissues. Since our results with syngeneic murine cancer model had indicated that in vitro cross-reactivity could be predictive of in vivo cross-reactivity, we did not pursue experimental studies with GH-pAb1.

To confirm the relevancy of this cross-cancer activity for patients, we evaluated their binding to patients’ biopsies (tissue microarray TMA]) from different cancer types. GH-pAb3 was confirmed to target melanoma with a binding to all patient biopsies for a mean stained area of 64.89% ± 5%. It also exhibited binding to non–small cell lung cancer (NSCLC) with 47.74% ± 4.94% of positive biopsies and, to a lesser extent, to colon with 22.18% ± 2.75% of positive biopsies (Table 2). GH-pAb2 was shown to target almost all liver patients’ biopsies, with an average of 8.31% ± 2.08%, but also almost all biopsies from anal cancer, head and neck cancer, and oesophageal cancer with a low intensity (1%–9% stained area) and 60% of gastric cancer of Asian descent, with an average of 45.32% ± 4.07% stained area on average on positive biopsies (Table 3).

Due to its positive staining on NSCLC patient biopsies, we evaluated GH-pAb3 antitumor activity in a human xenograft model of NSCLC. A549 cells were injected s.c. in the right flank of NMRI nude mice to generate a xenograft model of human NSCLC. I.p. administration of the treatment was started after the tumor became palpable (around 50 mm^3^) and continued twice a week for 4 weeks. Mice from the control group displayed a slow tumor growth from day 0, with median tumor size reaching 100 mm^3^ from day 20 and reaching 250 mm^3^ at the end of the protocol (day 30). Treated mice displayed significantly slower tumor growth compared with control, staying around 150 mm^3^ at the end of the protocol (day 30) (Figure 10D).

## Discussion

In this report, we show that oncolytic rabbit pAb raised against 3 different murine cancer type (HCC, SKCM, and CRC) present a target specificity with no avert recognition of nontumoral cells or normal tissues and with no obvious toxicity when repeatedly administered in vivo. However, this cannot be considered an absolute rule since, in another instance, an anti-4T1 rabbit pAb recognizes lung and brain tissues in IHC and presents an associated toxicity profile in vivo. The question whether heterologous pAb directed against tumor cell lines exhibit tissue specificity or not has not been addressed in earlier reports investigating their preclinical efficacy (15, 18, 20, 35), and we still lack data to state whether target tumoral tissue specificity is the rule or the exception. Nevertheless, preclinical and clinical lessons from rabbit pAb raised against human T lymphocytes mostly show that target specificity can be achieved with animal-derived pAb. Indeed, our results confirm that hyperimmunization with a given tumor cell line may elicit an immune response with a selective recognition of the targeted tumor cell and no binding to the corresponding healthy tissue. Furthermore, we showed in our studies that rabbit pAb raised against primary healthy murine hepatocytes showed a target specificity and were neither bound to nor affected by the viability of murine hepatocarcinoma tumor cells. This observation is analogous to these polyclonal responses against bacteria that are defined according to their serotype, inside a single species (36). To some extent, we could say that, in our experiments, rabbits immunized against mouse cell lines developed mouse serotypic-like tissue-specific antibody responses. A possible explanation is that shared antigens (such as MHC antigens) are less immunogenic in rabbits than lineage-specific antigens. This clearly warrants further investigations.

Our data demonstrate that rabbit oncolytic pAb exhibit several mechanisms of action to kill tumor cells in vitro: CDC, apoptosis, and recruitment of effector cells for ADCC or ADCP through engagement of activating Fcγ receptors (FcR). Fc-mediated effector cell recruitment and functions such as ADCC or ADCP probably play crucial roles for tumor-targeting antibodies in various animal models — an observation already published (15, 18, 20, 35). For example, the antibodies rituximab and trastuzumab lose their therapeutic activity in genetically modified mice that either lack expression of activating FcγR or are defective in FcγR signaling, while their efficacy is enhanced in FcγRIIb-KO mice (37, 38). On the other hand, the part of CDC in killing tumor cells in vivo is less clear, particularly for solid tumors, in part because tumor cells themselves express membrane-bound complement regulators (CD46, CD55, and CD59) (39). These regulators limit MAC formation and lysis of normal and cancer cells. In fact, these proteins are overexpressed in several tumor types, and their upregulation has been postulated to contribute to mAbs resistance in vivo (40).

In vivo, our data show that oncolytic pAb in the syngeneic tested models are as efficient or more efficient than, and potentially synergize with, immune checkpoint inhibitors like anti–PD-L1 antibodies. In the orthotopic HCC Hepa1.6 model, mice treated with pAb HCC or anti–PD-L1 mAb displayed a marked improvement of their clinical condition and a prolonged survival in combined therapy. However, while administration of pAb CRC in MC38 model led to a marked reduction in tumor growth, no such growth reduction was obtained with anti–PD-L1 mAb alone or in combination with the pAb, though MC38 cells express high level of PD-L1 (41). This might be explained by the lower dosing of anti–PD-L1 mAb compared with other in vivo studies in which a therapeutic response was observed (5 mg/kg versus 10 mg/kg) (42, 43). It is difficult to address the synergistic aspect with ICIs in the B16F10 model, due to the ulceration and necrosis of the tumors of the mice treated with anti–PD-L1 leading to their euthanasia for ethical reasons. However, the results obtained in the HCC demonstrate that a combination of our polyclonal oncolytic antibodies with ICIs is relevant and can maximize the antitumor effect.

Immune-excluded tumors and immune-desert tumors can be described as “cold tumors” and show low PD-L1 expression. In contrast to the inflamed phenotype, cold tumors rarely respond to ICI monotherapy. Interestingly, we observed that administration of pAb SKCM in B16F10 syngeneic model induced a T cell infiltrate in tumors of the melanoma mouse model, categorized as a cold tumor (44, 45), and none in tumors from untreated mice. Checkpoint inhibitor treatment, including blockade of the PD-1 receptor, has shown limited efficacy in the murine B16F10 melanoma model, despite strong expression of the ligand PD-L1 on the tumor cells (41) — a feature attributed to low tumor infiltration by effector CD8^+^ T cells (44, 45). A possible explanation of our observation is that pAb mediated CDC against target cells, releasing tumor-derived antigens and resulting in the local production of C3a and C5a anaphylatoxins that bind their respective GPCRs, C3aR and C5aR, expressed on T cells and antigen-presenting cells, and this binding drives T cell differentiation, expansion, and survival (46).

Even though tumor cells express tumor-associated antigens that can be recognized by cytotoxic T lymphocytes, they typically fail to induce a productive immune response. Their poor immunogenicity can be attributed to a variety of influences, including lack of T cell costimulation, loss of MHC class I expression, lack of MHC class II presentation to CD4^+^ Th cells, and production of immunosuppressive factors. Here, in addition to increased T cell infiltration, we observed de novo murine antitumor antibodies in MC38 and Hepa1.6 mouse models after pAb administration in mono- or bitherapy. Again, this might be due to the formerly described complement-induced T cell and APC activation (46). A large part of research in immunotherapy is based on strategies to boost antitumor CD8^+^ T cell–mediated response. The capacity of some antitumor pAb to trigger a humoral and cytotoxic response against tumoral cells might present clinical advantages since antibodies directed against tumors increase the lytic activity of intratumoral FcgR-expressing Th1 cells, which — in the absence of antibodies — may be inactive (47).

An unexpected finding in our study is the recognition by a single pAb of several tumor types, while it maintains absence of cross-reactivity toward normal cells and tissues. The anticancer activity of anti-CRC pAb was observed not only in a colon cancer model but also in a breast cancer model, while no toxicity was observed. Anticancer pAb can target several tumor-associated antigens simultaneously, which can explain multicancer efficiency and their effects on secondary metastatic cancers.

Beyond the complete inhibition of tumor growth following anti-CRC pAb administration, we also observed an abolition of lung metastasis in the 4T1 syngeneic model of breast cancer. This strong antimetastatic effect of oncolytic pAb was also confirmed in another pulmonary metastasis model where anti-B16F10 pAb drastically diminished the number of metastatic colonies in lungs. This activity possibly represents a strong advantage of pAb that are expected to deplete target cells first in the serum. Indeed, it seems accepted that CDC plays a central role in the elimination of circulating tumors in humans, even though this mechanism of action is controversial in the tumors (48). Several studies demonstrated that CDC is part of the mechanism of action of rituximab and ofatumumab (49, 50). The strong CDC activity of our pAb could explain the excellent therapeutic efficacy on migrating tumor cells and metastasis inhibition.

However, the main obstacle to the use of pAb in a clinic remains the high immunogenicity of pAb of animal origin. Indeed, humans differ from most mammals, with respect to 2 gene loss-of-function mutations that affect the shape of oligosaccharides, glycoproteins, and glycolipid: the Galactosyl-Transferase-1 (GT1) gene (51) encoding an α1-3-galactosyl transferase that catalyzes branching of galactose residues and the cytidine monophosphate acetyl hydroxylase (CMAH) gene (52, 53). A direct consequence is that Neu5Gc and Gal epitopes are excluded from “self-tolerance” and that natural anti-Neu5Gc and anti-Gal antibodies are present in humans (54). Infusion of antibodies from animal origin elicits strong immunogenicity, with side effects ranging from mild fever or skin rashes to more serious SSD (55, 56) or anaphylactic shock (57, 58). To circumvent these drawbacks and allow clinical evaluation of pAb in oncology, we generated GH-pAb, already shown to present an acceptable safety and tolerability profiles in humans (59, 60). Three new GH-pAb against 3 human cancer cell lines representative of colon adenocarcinoma, melanoma, and HCC were shown to exhibit strong anticancer activity in targeted primary tumor tissues and metastatic cancers, confirming the cross-cancer potential therapeutic benefit of the polyclonal approach. Neither binding to healthy tissues nor in vivo toxicity were observed. In fact, a careful preclinical exploration to eliminate any risk of cross-reactivity with healthy tissues and related toxicities, especially for tumors prone to metastasis, must be promoted. Our tissue cross-reactivity data suggest that immunohistology correlates with in vivo activity and therefore seems appropriate to mitigate this risk.

In conclusion, our study suggests, in agreement with other publications (15–20, 35, 61), that the therapeutic interest of pAb directed against tumor cells deserves to be revisited, now that engineered pAb with low immunogenicity become available (26, 57). Our data lay the groundwork for further development of oncolytic pAb, which may hold the potential to improve the fate of patients with metastatic solid cancers.

## Methods

### Animals and cell lines

Seven-week-old female C57BL/6 and Balb/c mice were obtained from Janvier Labs. New Zealand white rabbits were purchased from Hypharm. Double-KO pigs for CMAH and GGTA1 genes were housed in CER Group. Mouse cell lines included the following: Hepa1.6 (CRL-1830, murine HCC cells), MC38 (CRL-2868, murine colon adenocarcinoma cells), B16F10 (CRL-6475, murine melanoma cells), and 4T1 (CRL-2539, murine breast cancer), obtained from American Type Culture Collection (ATCC), and SK-MEL-30 (HTB-72, human melanoma), HCT-116 (CCL-247, human colon adenocarcinoma), Hep-G2 (HB-8065, human HCC), A549 (CRM-CCL-185, human NSCLC), and LNCaP (CRL-1740, human prostate cancer), purchased at DSMZ-Deutsche Sammlung von Mikroorganismen und Zellkulturen. All cells were cultured in DMEM supplemented with 10% FBS. Murine splenocytes were isolated and purified from murine spleens by perfusion with PBS 1×. Murine hepatocytes were isolated and purified as described elsewhere (62).

### Rabbit and pig immunization and polyclonal Ab preparation

The pAb were generated by immunizing the New Zealand white rabbits with tumor cells as previously described (18), with some modification. Briefly, rabbits were immunized with murine tumoral cells (hepatocarcinoma, colorectal, melanoma, and breast triple-negative cell lines) or with healthy murine hepatocytes (in 1 mL normal saline) by i.v. injection every 2 weeks for 8 weeks. One week after the fourth immunization, pAb was isolated using a Protein-A affinity chromatography system (AKTA Prime, GE) and kept at 4°C until used. Control antibody was similarly purified from whole normal rabbit serum of control animals. The GH-pAb were generated by immunizing double-KO–defined high–health status pigs with a human melanoma, colon adenocarcinoma, or HCC cell line. The swine IgG fraction was purified from serum in compliance with good manufacturing practice (GMP) and ICH guidelines. Details have been published elsewhere (26).

### In vitro assays

#### Binding on murine tumoral cell line or healthy splenocytes and hepatocytes.

In total, 250,000 cells were plated in conic 96-wells plate and incubated 30 minutes at 4°C with serial dilution of the corresponding purified pAb (maximal concentration 400 μg/mL following by 1:1.5 dilution on tumoral cell line and by 1:2 dilution on murine healthy splenocytes). After incubation with FITC-Prot A (1:250) for 30 minutes at 4°C, cells were analyzed by the BD FACSCanto Flow Cytometer (BD Biosciences). Plated hepatocytes were incubated with pAb mHp at 2 concentrations — 100 and 400 μg/mL — each for 30 minutes at 4°C. After incubation with Alexa Fluor 488–Protein A (1:250) for 30 minutes at 4°C, mean fluorescence intensity was analyzed using the NucleoCounter NC-3000 Advanced Image Cytometer (ChemoMetec A/S).

#### Complement dependent cytotoxicity assay.

In total, 100,000 cells were plated in V-bottomed 96-wells plate and incubated with serial dilution of the corresponding purified pAb (maximal concentration of 400 μg/mL following by 1:2 dilution) and rabbit complement (1:3 in RPMI) for 60 minutes at 37°C. At the end of the incubation period, propidium iodine was added and cells were analyzed by the BD FACSCanto Flow Cytometer (BD Biosciences). To evaluate CDC in primary murine hepatocytes and Hepa1.6 tumor cells, serial dilution of pAb HCC or pAb mHp (maximal concentration, 100 μg/mL) was incubated with hepatocytes and rabbit complement (1:6 in maintenance medium) for 24 hours at 37°C. At the end of the incubation period, supernatants were analyzed using the LDH cytotoxicity kit according to the supplier’s protocol (Roche). Plates were then read out by optical density (TECAN).

#### Apoptosis assay.

Murine cancer cell lines or healthy splenocytes were treated with increasing doses of purified IgG in RPMI medium with 10% FCS (both from Thermo Fisher Scientific). Nonimmune IgG was used as a negative control. After 3 hours of culture (37°C, 5% CO_2_), cells were labeled with Alexa Fluor 488–conjugated annexin V and DAPI (Thermo Fisher Scientific) before analysis on a CELESTA flow cytometer (BD Biosciences). Percentages of cells in early apoptosis (annexin V^+^/DAPI^–^ cells) and in late apoptosis (annexin V^+^/DAPI^+^) were combined to determine the overall percentage of apoptosis.

#### Antibody-dependent cell-mediated cytotoxicity.

Murine splenocytes were isolated from normal spleens of C57BL/6 mice. Murine splenocytes were directly used in binding assays or prepared to isolate NK Cells according to the MojoSort Mouse NK Cell Isolation Kit protocol (BioLegend). The day before the assay, murine tumor cell lines (Hepa1.6, B16F10, and MC38) were labeled with CFSE (Invitrogen) and cultured in RPMI 10% FCS to be adherent the day of the assay. The day of the assay, oncolytic rabbit pAb were added at a concentration of 5 μg/mL and cocultured with murine NK (effector/target [E:T] ratio, 4:1) in RPMI 10% FCS for 16–24 hours. After 16–24 hours of incubation, propidium iodine was added and cells were analyzed (CFSE^+^/PI^+^ cells) by the NucleoCounter NC-3000 Advanced Image Cytometer (ChemoMetec A/S).

#### Antibody-dependent cell-mediated phagocytosis.

Phagocytosis rate was evaluated with mouse BM-derived macrophages (BMDM). BM cells were collected from the femur and tibia of mice as described elsewhere and then differentiated in macrophages by adding M-CSF1 in culture medium (1 ng/mL final). After 7 days of culture, macrophages were labeled with mouse F4/80 Alexa Fluor 647 conjugate (Invitrogen). The day before the assay, murine tumor cell lines (Hepa1.6, B16F10, and MC38) were labeled with CFSE (Invitrogen) and cultured in RPMI 10% FCS to be adherent the day of the assay. The day of the assay, oncolytic rabbit pAb were added at a concentration of 5 μg/mL and cocultured with labeled macrophages (E:T ratio, 4:1) in RPMI 10% FCS for 16–24 hours. After 16- to 24-hour incubation, cells were trypsinized and were analyzed (CFSE^+^/Alexa Fluor 647^+^ cells) by the NucleoCounter NC-3000 Advanced Image Cytometer (ChemoMetec A/S)

#### Mouse anti-HCC and anti-CRC IgG detection in mouse serum.

Target murine cells (Hepa1.6 or MC38) were coated into ELISA plates in NaH_2_PO_4_ coating buffer (0.2M; 2 hours at 37°C). After washing, saturation was obtained by incubating the coated cells 1 hour at room temperature with PBS 0.05%, Tween 20, and BSA 2%. Mouse serum was incubated 1 hour at room temperature, diluted in PBS 0.05%, Tween 20, and BSA 2%. After washing, mouse anti-HCC or anti-CRC IgG were detected with HRP-conjugated anti-mouse IgG diluted 1/1,000 in PBS 0.05%, Tween 20, and BSA 2%; incubated 1 hour at room temperature; and washed and revealed with TMB reagent. Optical density was read at 450 nm with a correction at 630 nm (TECAN).

### In vivo experiments

#### Syngeneic mouse tumor models.

A mouse model of HCC was obtained by an intraportal injection of 250,000 Hepa1.6 murine HCC in C57BL/6 mice. Mouse models of melanoma and colorectal cancer were obtained by s.c. injection of 250,000 B16F10 cells (murine melanoma cells) and MC38 cells (murine colorectal cells), respectively, in the right flank of C57BL/6 mice. Mouse orthotopic model of breast cancer was obtained by injection of 4T1 (murine breast cancer cells line) in the mammary gland of female Balb/c mice. Treatment was initiated from day 4 after surgery and occurred twice a week for a total duration of 28 days. Treatment consisted on the i.p. injection of a control isotype 3G8 (nonspecific mAb) at 5 mg/kg and nonimmune rabbit IgG at 12.5 mg/kg for group “Control” (*n* = 7); control isotype 3G8 at 5 mg/kg and oncolytic pAb at 12.5 mg/kg for the treated group with monotherapy (*n* = 8); anti–PD-L1 monoclonal Ab (clone 1F.9G2, BioXCell) at 5 mg/kg and oncolytic pAb at 12.5 mg/kg for the treated group with bitherapy (*n* = 8); and anti–PD-L1 monoclonal Ab at 5 mg/kg and nonimmune rabbit IgG at 12.5mg/kg for group “mAb PD-L1” (*n* = 7).

HCC evolution was evaluated by recording survival rate and cancer clinical score progression (from 0 to 4 with 0 indicating normal behavior and appearance; 1 indicating bristly hair, slightly swollen abdomen; 2 indicating slightly swollen abdomen, oedema; 3 indicating swollen abdomen, exophthalmia; and 4 indicating swollen abdomen (diam > 9cm), prostration). When clinical score 4 was reached, euthanasia of the mouse was performed for ethical purpose. For the other syngeneic s.c. or orthotopic models, the tumor growth was assessed by measuring tumor size.

#### Pulmonary metastatic model.

Syngeneic in vivo mouse model of pulmonary metastasis was obtained by i.v. injection (tail vein) of 250,000 B16F10 murine melanoma cells in C57BL/6 mice. Treatment was initiated from day 1 after surgery and occurred twice a week for 14 days. At day 14, mice were sacrificed and lungs were observed to detect the presence of nodules, easily identifiable by the presence of melanin. The control group did not receive any treatment (*n* = 10), while the other group (*n* = 10) received treatment consisting of i.p. injection of pAb SKCM at 25 mg/kg. After sacrifice, lungs were harvested and cleaned by PBS washing. Black nodules in the lung were counted by macroscopic examination.

#### Human tumor xenograft models.

In total, 1 × 10^6^ cells of each human cell line — HCT116, SK-MEL-30, Hep-G2, A549, and LNCaP — were injected s.c. in the right flank of NMRI nude mice to generate 5 human cancer models: colon adenocarcinoma, melanoma, HCC, NSCLC, and prostate cancer, respectively. Treatment with GH-pAb was initiated when tumor size reach 50 mm^3^ and occurred twice a week for 4 weeks at a dose of 35 mg/kg by i.p. route.

#### Tumor growth measurement.

By using a caliper, we measured width (W) and length (L) of the tumor and then calculate the volume using a standard formula: 1/2 × W × W × L, thus assuming a standard and constant shape of the tumor. Growth of the tumor in height was not measured, since only W and L changes will affect the calculated volume.

### Immunohistological analyses and quantification of TMA

Lung, brain, striated skeletal muscles, intestine, kidney, liver, and heart tissues were collected after sacrifice of normal mice and embedded in OCT compound in cryomolds. Oncotest PDX tumor TMA slides were purchased at Charles River Discovery Research Services.

Primary antibodies used in this study were rabbit and swine pAb and were used at a concentration of 5μg/mL, anti-CD3 (1:100; BD Biosciences), anti-CD8 (1 μg/mL; Thermo Fisher Scientific). For fluorescence labeling, we used secondary antibodies conjugated to Alexa Fluor 488 (1:500; Invitrogen). For bright-field microscopy, we used biotinylated goat anti-rabbit secondary antibody with HRP-conjugate (1:200; Jackson ImmunoResearch) or goat anti-pig secondary antibody with HRP-conjugate (1:1,000; Mabtech AB) stained with ImmPACT VIP Substrate Kit (Vector Laboratories).

### Statistics

Flow cytometry data were analyzed with FlowJo. Statistical analyses were done with Prism 8 (GraphPad Software) and XLStat software. Kaplan-Meier curves were analyzed with the log-rank test. Log-rank test and χ^2^ test were used for comparison of control groups and treated groups in hepatocarcinoma model and breast cancer model. One-way ANOVA with Newman-Keuls or Fisher post hoc test was used for comparison of control groups and treated groups in colon adenocarcinoma model. Two-tailed Student’s *t* test was used for comparison of control groups and treated groups in pulmonary metastasis models and human xenograft tumor models. TMA quantifications were made with ImageJ software (NIH).

### Study approval

All procedures involving the use of animals in this study were carried out in accordance with EU directive 2010/63/EU regulating animal experimentation after authorization by relevant authorities: APAFIS 19472, APAFIS 29523, APAFIS 26124.

### Data availability

The data that support the findings of this study are available from the corresponding author upon reasonable request. Values for all data points in graphs are reported in the Supporting Data Values file.

## Author contributions

BV, FB, CC, and OD conceived the study. BV and CC designed and supervised some experiments. CC, PM, JR, PR, OG, SB, and PJR performed the experiments. BV and CC analyzed data.

## Supplementary Material

Supplemental data

Supporting data values

## Figures and Tables

**Table 3:**
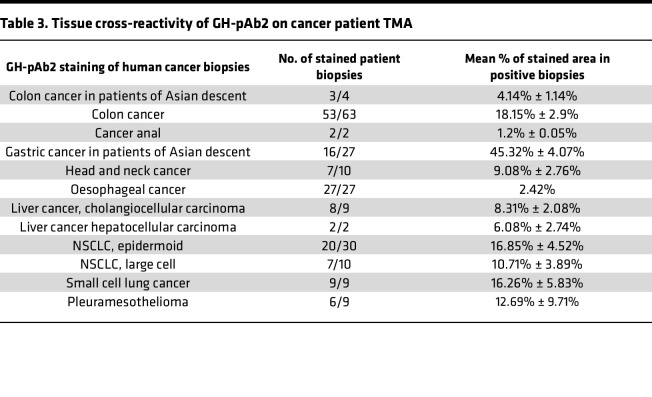
Tissue cross-reactivity of GH-pAb2 on cancer patient TMA

**Table 2:**
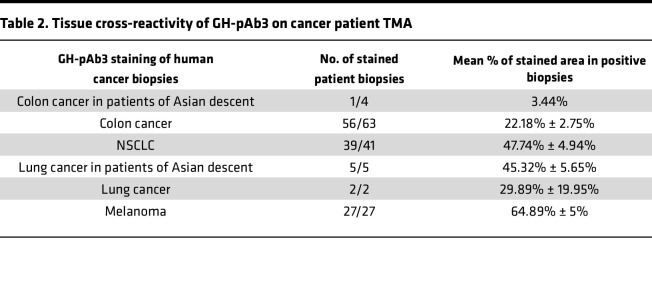
Tissue cross-reactivity of GH-pAb3 on cancer patient TMA

**Table 1:**
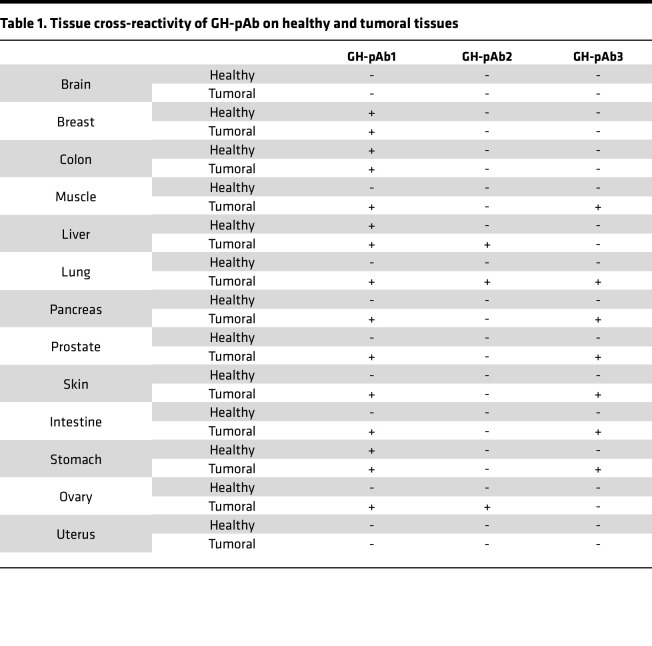
Tissue cross-reactivity of GH-pAb on healthy and tumoral tissues

## Version 1 12/12/2023

In-Press Preview

## Version 2 02/08/2024

Electronic publication
