# Supplementary material for: Polyclonal antibodies selectively inhibit tumor growth and invasion and synergize with immune checkpoint inhibitors
Source: JCI Insight. 2024 Feb 8;9(3):e166231. doi: 10.1172/jci.insight.166231 (PMC10967460; doi:10.1172/jci.insight.166231)
Supplement: Supplemental data [file jciinsight-9-166231-s261.pdf]

Supplementary figures

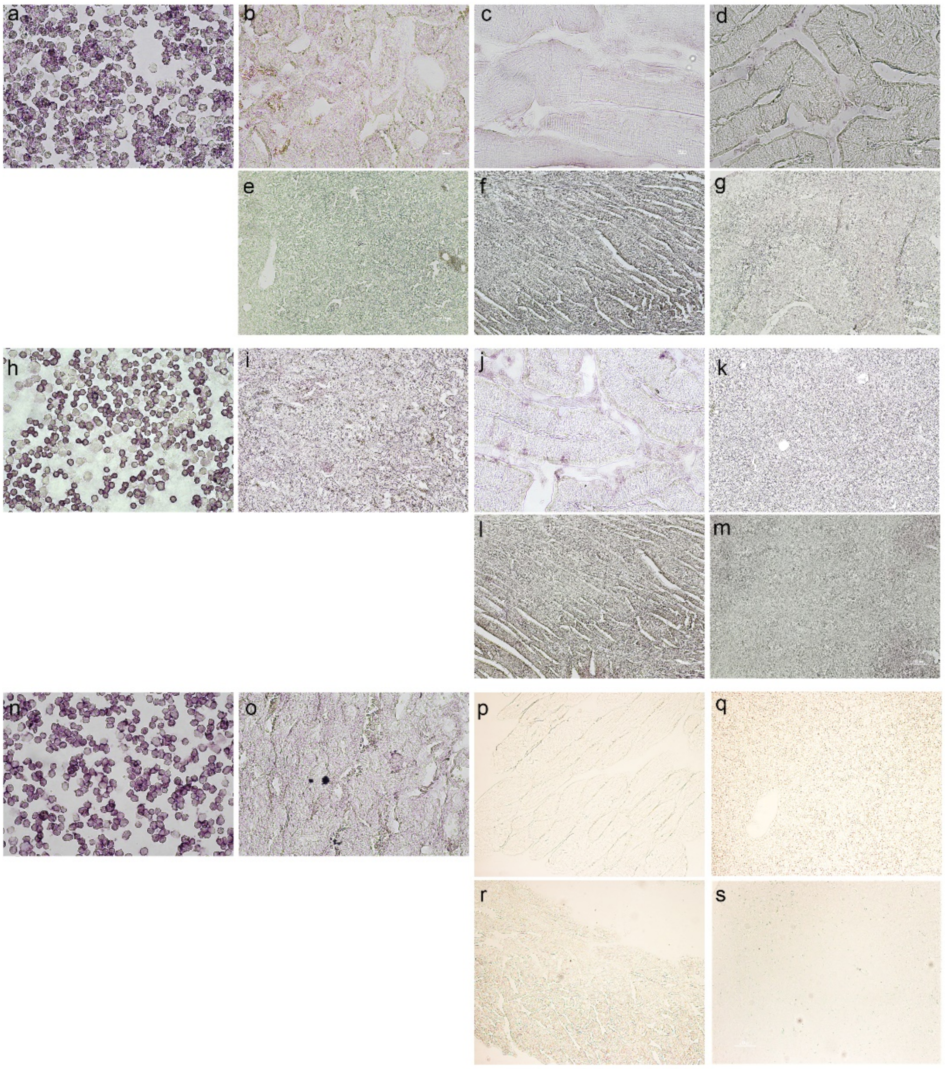

Figure S1 Absence of cross-reactivity in healthy murine tissues with pAb CRC, pAb HCC and pAb SKCM

(a-g) Immunostainings with pAb CRC at a concentration of 5 µg/m in tumor cells (positive control) and in various healthy tissues. Strong staining is observed in MC38 cells (a) while no staining is reported in kidney (b), striated skeletal muscles (c), intestine (d), liver (e), heart (f), central nervous system (g). (h-m) Immunostainings with pAb HCC at a concentration of 5 µg/m in Hepa1.6 cells (positive control) and in various healthy tissue. Strong staining is observed in Hepa1.6 cells (h) while no staining is reported in kidney (i), intestine (j), liver (k), heart (l), central nervous system (m). (n-s) Immunostainings with pAb SKCM at a concentration of 5 µg/m in tumor cells (positive control) and in various healthy tissues. Strong staining is observed in B16F10 cells (n) while no staining is reported in kidney (o), striated skeletal muscle (p), liver (q), heart (r) and central nervous system (s).

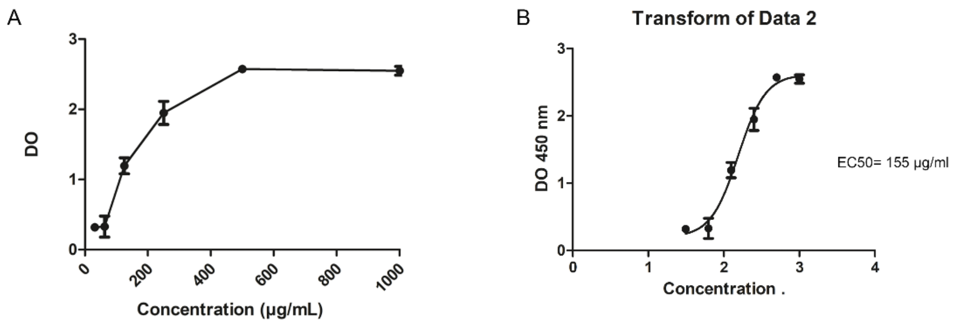

Figure S2 pAb TBNC strongly targets the metabotropic glutamate receptor 1 (mGlur1)

mGlur1 is coated on a Maxisorp (NUNC) ELISA plate at 100 ug/ml overnight at 4°C in carbonate buffer at PH 9.5. After saturation and washing, increasing concentrations of pAb TBNC (31.25, 62.5, 125, 250, 500 and 1000 µg/ml) are added and incubated for 1 hour at room temperature. After washing, HRP-Prot A antibody (Invitrogen, Waltham, Massachusetts, United States) is added at a dilution of 1:10 000 and incubated for 1 hour at room temperature and visualized with tetramethylbenzidine (TMB) reagent. Optical density is read at 450 nm. pAb TBNC targets and binds the metabotropic glutamate receptor 1 (mGlur1) with an EC50= 155 µg/ml.
